# *In vivo* evaluation of the effect of lithium on peripheral circadian clocks by real-time monitoring of clock gene expression in near-freely moving mice

**DOI:** 10.1038/s41598-019-47053-3

**Published:** 2019-07-29

**Authors:** Yuka Sawai, Takezo Okamoto, Yugo Muranaka, Rino Nakamura, Ritsuko Matsumura, Koichi Node, Makoto Akashi

**Affiliations:** 1 The Research Institute for Time Studies, Yamaguchi University, 1677-1 Yoshida, Yamaguchi, 753-8511 Japan; 2 Department of Cardiovascular Medicine, Saga University, 5-1-1 Nabeshima, Saga, 849-8501 Japan

**Keywords:** Gene expression, Neurophysiology

## Abstract

Lithium has been used as a mood stabilizer to treat human bipolar disorders for over half a century. Several studies have suggested the possibility that the efficacy of lithium treatment results in part from the amelioration of circadian dysfunction. However, the effect of lithium on clock gene expression has not yet been investigated *in vivo* because continuous measurement of gene expression in organs with high time resolution over a period of several days is difficult. To resolve this issue, we attached a small photo multiplier tube (PMT) tightly to the body surface of transgenic mice carrying a reporter gene such that the photon input window faced target organs such as the liver and kidney and succeeded in long-term continuous measurement of circadian gene expression in semi-freely moving mice over periods of several weeks. Using this simple method, we clearly showed that lithium causes circadian period elongation in peripheral clock gene expression rhythms *in vivo*. Further development of our detection system to maturity will aid a wide range of research fields in medicine and biology.

## Introduction

Several studies have suggested the possibility that mood stabilizing effects of lithium result in part from the amelioration of circadian dysfunction^1,2^. One piece of evidence for the putative action of lithium on circadian rhythms is that lithium administration lengthens the circadian period in behavior and physiology^3^. In addition to this behavioral and physiological effect, administration of lithium to cultured cells and tissues causes circadian period elongation in clock gene expression rhythms^4–6^, suggesting that lithium directly acts on cell-autonomous core circadian machinery *in vitro* and *ex vivo*. However, a significant question is why the medium concentrations of lithium required for circadian period elongation in clock gene expression rhythms *in vitro* and *ex vivo* are much higher than the plasma concentrations required for period elongation in behavioral rhythms *in vivo*^5^. The effect of lithium on circadian period of clock gene expression has not yet been investigated *in vivo*.

Detection of changes in period length of clock gene expression rhythms *in vivo* using traditional experimental approaches is technically difficult. Obtaining biochemical data on sequential changes in gene expression over a period of a few days requires extensive experimentation: for example, if organs are harvested 16 times with a sampling interval of 3 hours over a period of 48 hours and measurements are done in triplicate, the total number of mice used will be 48. Following this overnight organ collection, vast numbers of frozen organs are individually homogenized, and gene expression levels are quantified by biochemical analysis. Despite this extensive experimentation, the 3-h interval expression data thereby obtained are not sufficient for reliable evaluation of circadian period length because of low time resolution and short measurement period. Increasing sampling frequency and extending the sampling period may solve this problem, but it is time- and cost-prohibitive. Some of the technical limitations mentioned above can be solved with *in vivo* imaging technology^7^. Photons from anesthetized mice carrying the *luciferase* gene, the expression of which is controlled by a target gene promoter, are detectable with a high-sensitivity camera as relative expression levels of the target gene. However, images must be taken at set time points overnight, which limits efforts to extend the measurement period and increase time resolution by narrowing measurement intervals. In addition, repeated exposure to anesthesia may have a marked impact on animal physiology.

In the present study, to investigate the effect of lithium on circadian period length of clock gene expression with high time resolution *in vivo*, we aimed to monitor the sequential expression of circadian clock genes in real time. A few studies have reported real-time monitoring of bioluminescence driven by a clock gene promoter in animal organs. For example, Yamaguchi *et al*. reported a fascinating method for real-time detection of bioluminescence emitted from the hypothalamic suprachiasmatic nuclei (SCN), in which an optical fiber was inserted into a mouse brain^8^. However, this method requires a specific technique, and whether or not long-term monitoring using this method is possible remains unclear, as the brain tissue is partially but severely damaged. On the other hand, a recent study reported the real-time monitoring of liver clock gene expression in freely moving mice in dark boxes equipped with a highly sensitive camera or photo multiplier tube (PMT)^9^. This method overcomes many of the problems mentioned above and allows real-time monitoring in the liver for up to 10 days. In addition to this measurement method, a very recent study reported another *in vivo* imaging technique to perform long-term imaging of circadian gene expression by using dual-focal 3D tracking^10^. However, these two systems would be too cumbersome and expensive. In the present study, we therefore aimed to develop a simple and low-cost method for real-time monitoring of clock gene expression rhythms *in vivo*.

## Materials and Methods

### Animals

*Period2*^*luciferase*^ (*Per2*^*Luc*^) knock-in mice and *Bmal1-Eluc* (enhanced green-emitting luciferase) transgenic mice were gifts from Dr. Joseph Takahashi and Dr. Yoshihiro Nakajima, respectively^11,12^. Animals were bred and maintained on a 12-hour light-dark (LD) cycle (lights on at 9:00 A.M.) and allowed *ad libitum* access to food and water. Real-time monitoring of bioluminescence was done using male C57BL/6 background *Per2*^*Luc*^ and *Bmal1-Eluc* mice depilated of body hair around target organs with depilatory cream or male C57BL/6 background *Tyrosinase* mutant (*Tyrc-Brd*) homozygous *Per2*^*Luc*^ mice. Pigment is completely absent from skin, hair and eyes in mice homozygous for *Tyr*. The number and age of mice used in each experiment are indicated in the figure legends. All protocols for animal experiments were approved by the Animal Research Committee of Yamaguchi University. Animal studies were performed in compliance with the Yamaguchi University Animal Care and Use guidelines.

### *In vivo* imaging

The Lumazone CMS imaging system (NipponRoper, Tokyo, Japan) was utilized for *in vivo* imaging. Mice were injected with *in vivo*-grade luciferin (VivoGlo Luciferin, *in vivo* grade; Promega, Fitchburg, WI, USA) s.c. on the back near the neck at a dose of 8.3 mg/kg. Images were then taken at 5 min after luciferin injection with 5-min exposure from the dorsal aspect under isoflurane anesthesia inside a dark box.

### PMT method

Real-time detection of bioluminescence was performed by attaching a PMT (R7400P/E5780; Hamamatsu Photonics, Hamamatsu, Japan) onto the body surface. The photon input window of the PMT directly and tightly faced the target organ. In this experimental approach, the distance from the PMT to the target organ was not only nearly constant but also as short as possible, and ultraweak bioluminescence therefore became detectable without the need for enhancing signals by strong overexpression of luciferase. The weight of the PMT was about 10 g, the physical stress was therefore reduced by elastically hanging it from a top board of the dark box. Although tight attachment of the PMT to the body surface prevented the mice from moving with complete freedom, this was considered beneficial for monitoring, as any vigorous movements caused data noise due to vibrations. Mice were able to access food and water *ad libitum*.

Detailed information on measurement procedures is as follows. Black mice can be used if body hair around the target organs is depilated but are not suitable for long-term monitoring of bioluminescence because body hair regrows in a week. Luciferin (*in vivo* grade, 157 mM) was continuously supplied from an osmotic pump (2001, ALZET) implanted intraperitoneally or subcutaneously with a release rate of 1 microliter per hour and a release duration of up to one week, according to manufacturer’s instructions. However, to enhance bioluminescence and extend the measurement period, a syringe pump (YSP-201; YMC, Kyoto, Japan) was used for continuous subcutaneous supply of luciferin (*in vivo* grade; Promega). The release rate of luciferin (7.85 mM) was 30 µL/h. After operation for subcutaneous insertion of a silicone tube (BC-5S/5Fr, Access Technologies, Skokie, IL, USA) under continuous anesthetization with vaporized isoflurane (1.8–2.8%), mice were jacketed to ensure that the PMT was tightly attached to the body surface. Specifically, an open cylinder made of sponge-like material was fixed as a socket for the PMT on the mouse’s back with an elastic medical band. When the kidney was a target organ, these were localized around the lowest rib on the back, where the center of the cylinder was set. The cables and silicone tube were covered with a hard plastic protector. Although photon counting was initiated under constant dark conditions immediately after these manipulations and surgical operation, data for the first 2 days were not used for circadian analysis, given the need for recovery from surgical damage, habituation to the measurement environment and stabilization of the blood concentration of luciferin.

Mice were separated and kept singly in a dark box (349[W] × 468[D] × 368[H] mm) specified for photon counting. The back wall of the box was doubled, and each layer had a ventilation hatch. The hatches were localized away from each other to avoid the entry of outside light. To further reduce background noises to detect ultraweak photon emission, a third ventilation hatch was installed at the end of a hairpin-curved air duct. Continuous real-time photon counting was performed with a counting unit (C11859; Hamamatsu Photonics, Hamamatsu, Japan). Peak times of bioluminescence were calculated by cosine fitting and plotted as a function of time. To assess the presence or absence of significant circadian rhythmicity in bioluminescence data, we performed a cosine-fitting procedure and calculated the index of goodness of fit, with smaller index values reflecting a better-fit data set. To calculate this index, expression data were analyzed with the software Acro. P-values of < 0.05 were considered statistically significant. To calculate the average circadian period length based on several-day bioluminescence data, cosinor analysis was performed using the Cosinor software. Both of the Acro and Cosinor software were kindly provided by Dr. Refinetti (https://www.circadian.org/softwar.html).

### *Ex vivo* tissue culture

Mice were sacrificed and used to prepare kidney explants. Slices of 300 μm thickness were prepared from kidney tissue using a McIlwain Tissue Chopper in ice-cold HBSS (#14025, Life Technologies, CA) supplemented with 10 mM HEPES. Each tissue slice was placed onto a Millicell insert (#PICMORG, Merck Millipore, MA) in a 35 mm dish and cultured in DMEM (#D2902, Sigma, MO) supplemented with 0.035% sodium bicarbonate, 10 mM HEPES, 4.5 g/L D-glucose, 1.0% penicillinstreptomycin and 10% FBS. After preculture for approximately 48 h, all explants were treated with 100 nM dexamethasone (Dex) for 60 min, and cultured in fresh DMEM containing 0.1 mM D-Luciferin (Promega, WI) and different concentrations of lithium. Bioluminescence was measured at 36 °C and integrated for 1 min at intervals of 15 min using an LM2400 (Hamamatsu Photonics, Japan). For statistical analysis, data were detrended by subtracting the 24-h moving average from raw data. The value of the first peak was regarded as 100 (time = 0). Cosinor analysis was performed using the software Cosinor.

### Adenovirus infection

An adenovirus carrying the *luciferase* gene driven by circadian promoter/enhancer elements was constructed using the ViraPower Adenoviral Gateway Expression Kit (K4940-00, Thermo Fisher Scientific). Together with the *luciferase* gene, the transcription-regulatory region of the *hBmal1* gene was excised from the previously constructed vector *hBmal1* (−3465–+57) -pGL3^13^ and the insert was subcloned into the pENTR-1A vector. The resulting vector *hBmal1* (−1683–+57) -*luc*-pENTR-1A was used for recombination with the pAd/PL-DEST vector. The resulting pAd/PL-DEST vector was transfected into HEK293A cells to produce an adenovirus vector carrying *hBmal1-Luc*. The adenovirus was amplified and purified using the Vivapure AdenoPACK20 Kit (VS-AVPQ020, Sartorius), and 1 × 10^11~12^ viral particles were then infected to mice by tail vein injection. Successful luciferase expression was confirmed by *in vivo* imaging 24 h after infection.

## Results

### A PMT method enables simple monitoring of *in vivo* gene expression

**Figure 1:**
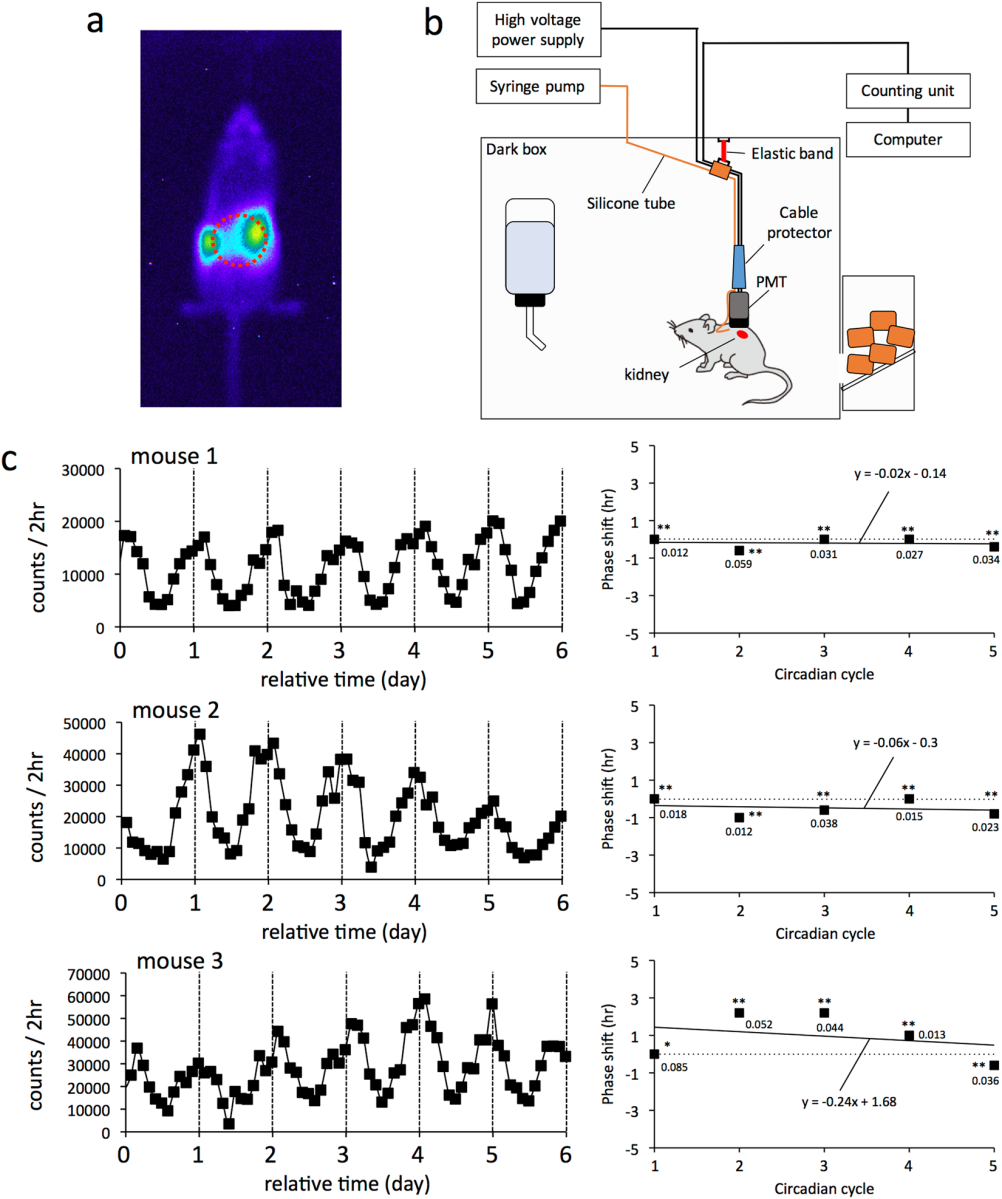
A PMT method for long-term real-time monitoring of *in vivo* gene expression. (**a**) The Lumazone CMS imaging system was used for *in vivo* imaging. Mice were injected with luciferin subcutaneously. Images were taken from the dorsal aspect under isoflurane anesthesia inside a dark box. (**b**) A schematic representation of a PMT method for long-term real-time monitoring of *in vivo* gene expression. After operation for subcutaneous insertion of a silicone tube under continuous anesthetization, mice were jacketed to ensure that the PMT was tightly attached to the body surface. The photon input window of the PMT directly and tightly faced the kidney area. Physical stress was reduced by elastically suspending the PMT from the top board of the dark box. If mice gnaw the high voltage power supply cable, they will die from electrocution. To avoid this emergency, the cable was covered with a hard-plastic protector. Although this jacketing prevented the mice from moving with complete freedom, they were able to access food and water *ad libitum*. To permit long-term measurement and enhance bioluminescence, a syringe pump was used for continuous subcutaneous supply of luciferin. Bioluminescence was measured under constant dark conditions. (c, left) Data show the two-hour integrated number of photons and circadian oscillation in bioluminescence emitted from the kidney in three individual mice. Data are from three representative male mice. (c, right) Circadian phases of bioluminescence data obtained from three individual mice were calculated by cosine fitting and plotted as square dots. The first circadian phase was defined as 0. Statistically significant circadian cycles are marked with asterisks (*P < 0.005; **P < 0.001). Values near squares indicate goodness-of-fit for each circadian cycle. Regression analysis was performed on sequential phase shifts, and the regression formula and line are shown in each graph.

In this study, we utilized mice expressing the circadian protein Period2 fused with firefly luciferase (*Per2*^*Luc*^ mice) to develop a detection system for long-term real-time monitoring of gene expression in animal organs^11^. Autonomous and cosine-like oscillation of clock gene expression is at the center of the molecular machinery driving the circadian clock. As the goal of the present development, we therefore aimed to obtain long-term circadian oscillation data from mice with nearly free movement. The circadian clock is cell-autonomous, and clock gene expression therefore oscillates in every cell except for a few tissues^14,15^. *In vivo* imaging data of the mouse’s dorsal side identified strong signals from the kidney (Fig. 1a), which was therefore suitable as a target organ for developing a new detection system in the present study. It is generally known that the anatomical positions of the left and right kidneys differ slightly, and this may be the reason why bioluminescence intensity was different between them. Using the same *Per2*^*Luc*^ mice as those used in the present study, a previous study with an *in vivo* imaging technique found a positive correlation between the amount of luciferin and bioluminescence intensity from the kidney^7^. This study also confirmed that bioluminescence from the dorsal side was eliminated after artery ligation of the kidney, providing strong evidence that the dorsal bioluminescence was from the kidney.

Real-time detection of bioluminescence was performed by simply securing a small PMT tightly to the body surface so that the photon input window of the PMT directly faced the kidney area (Fig. 1b). We fixed an open cylinder made of sponge-like material as a socket for the PMT on the mouse’s back with an elastic medical band (Fig. S1). The kidneys are localized around the lowest rib on the back, where the center of the cylinder was set. In this experimental approach, the distance from the PMT to target organs was not only nearly constant but also as short as possible. Although the weight of the PMT was about 10 g, the physical stress was reduced by elastically suspending the weight from the top board of the dark box (see Fig. 1b). While the mice were jacketed to fix the PMT and could not move with complete freedom, they were able to access food and water *ad libitum*. Although a previous study indicated that circadian rhythms in bioluminescence from the mouse liver are detectable *in vivo* simply by dissolving luciferin in drinking water^9^, we failed to detect circadian bioluminescence using this supply method in our measurement system. This might have been because blood concentrations of luciferin were inconstant, probably due to diurnal variation in water drinking. Many previous studies have reported that circadian bioluminescence in peripheral and central clocks is detectable *in vivo* by supplying luciferin using an osmotic pump^16–20^. We therefore implanted an osmotic pump intraperitoneally or subcutaneously in mice to provide a continuous supply of luciferin. However, the release duration was limited to a maximum of 1 week in accordance with the manufacturer’s instructions. It is unfortunately difficult to replace an empty pump with a filled one without disturbing the constant dark conditions. Moreover, this surgical operation during measurement may affect experimental results. To extend the measurement period without pausing and disturbing measurement, we therefore replaced the osmotic pump with a syringe pump, which allowed the uninterrupted subcutaneous supply of luciferin. Thus, our method requires only a simple manipulation to provide real-time and long-term monitoring of gene expression: after a simple operation to subcutaneously insert a silicone tube, all the researcher has to do is jacket the mouse to ensure tight attachment of the PMT to the body surface.

Three example data obtained with the PMT method show clear circadian oscillation of bioluminescence (Fig. 1c, left). The peak time in PER2 protein levels was biochemically reported to be around ZT15–18^21^, which appeared to be consistent with the phase in the bioluminescence data obtained by the PMT method. The period length was confirmed to be constant during measurement by calculating peak times (Fig. 1c, right). Significant circadian cycles were defined by cosine fitting with Acro, a software application provided by Roberto Refinetti, and measurements were defined as success when significant circadian cycles continued over the period of more than three days. The success rate of the PMT method was good: at this time, the success rate for detecting significant circadian oscillation was more than 95% of measurements for a period of more than three days and more than 80% of measurements for a period of more than five days, and the maximum successful monitoring period is more than six weeks. There are several causes for measurement failure: in many cases, mice escaped from the PMT jacket, while in others the silicone tube for subcutaneous luciferin supply was bitten off or pulled out of the back. In these cases, there was no alternative but to give up the measurement because fixing the failure under dark conditions was not only difficult but also disturbed the experimental results.

### Evaluation of the reliability of the PMT method

**Figure 2:**
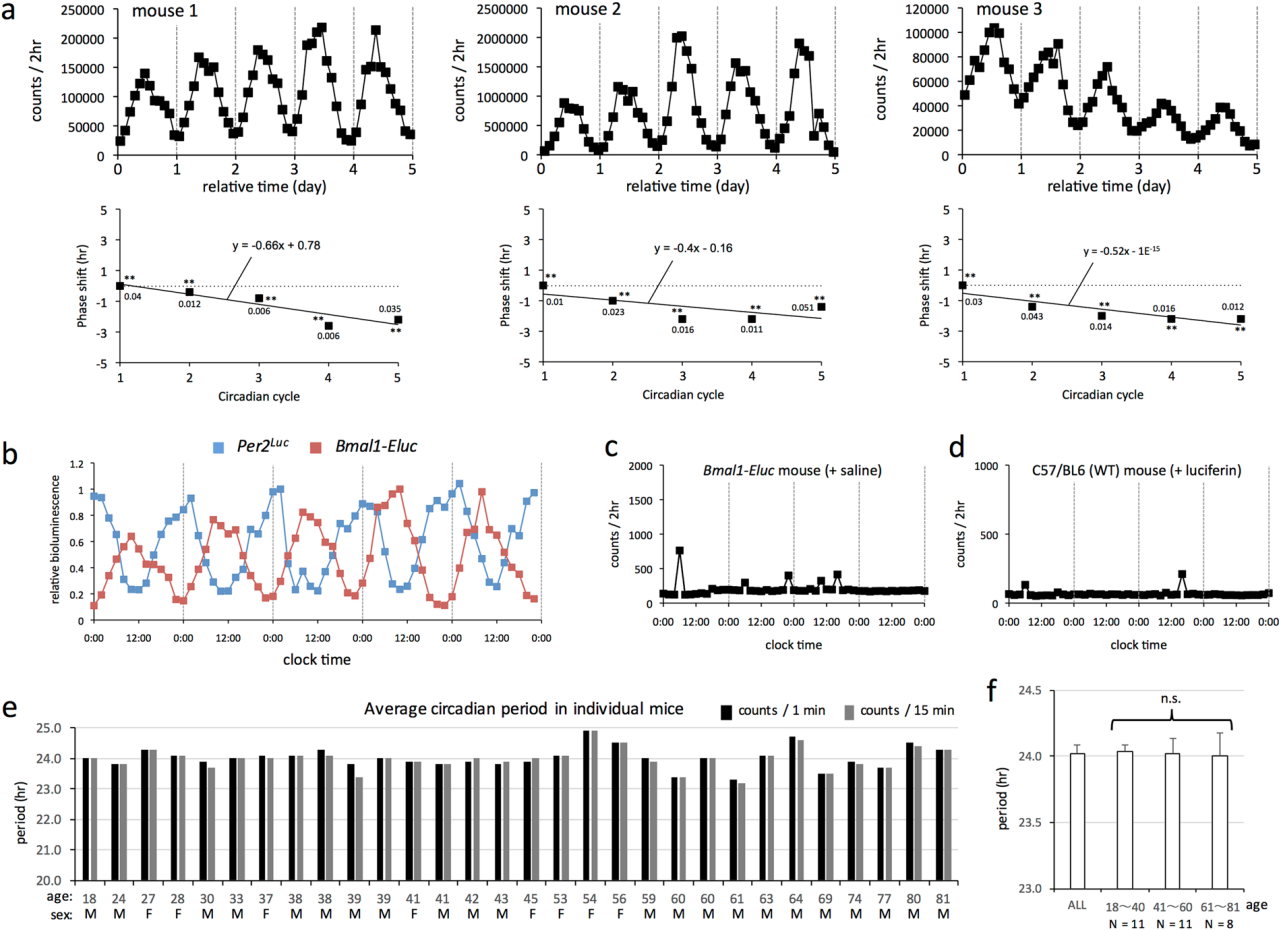
Evaluation of the reliability of the PMT method. (a, top) Real-time *in vivo* monitoring of bioluminescence was performed using *Bmal1-Eluc* mice. After subcutaneous insertion of a silicone tube for continuous supply of luciferin, mice were jacketed to ensure that a PMT was tightly attached to the kidney area. Mice were kept individually in a dark box specified for photon counting, with *ad libitum* access to food and water. Data show the two-hour integrated number of photons emitted from the kidney area for three individual mice. (a, bottom) Circadian phases of bioluminescence data obtained from three individual mice were calculated by cosine fitting and plotted as square dots. The first circadian phase was defined as 0. Statistically significant circadian cycles are marked with asterisks (*P < 0.005; **P < 0.001). Values near squares indicate goodness-of-fit for each circadian cycle. Regression analysis was performed on sequential phase shifts, and the regression formula and line are shown in each graph. (**b**) The data of mouse #1 (*Bmal1-Eluc*) in (**a**) were merged with those of mouse #1 (*Per2*^*Luc*^) in Fig. 1c. To facilitate comparison of phase difference between these two mice, the maximum value of integrated bioluminescence was set to 1. (**c**,**d**) Background and noise were evaluated using real-time *in vivo* monitoring of bioluminescence. A PMT was tightly attached to the kidney area of *Bmal1-Eluc* mice continuously supplied with plain saline (**c**) and wild-type mice continuously supplied with luciferin-containing saline (**d**). (**e**) Based on seven-day bioluminescence data obtained from individual *Per2*^*Luc*^ mice using the present method, the average circadian period length was calculated by cosinor analysis. Black and gray bar graphs indicate the average period length calculated based on photon counts per 1 and 15 minutes, respectively. (**f**) Mice in (**e**) were divided into three groups based on age, and one-way ANOVA analysis was performed to examine the effect of aging on period length. N.S. represents “no significant difference”.

Although we succeeded in long-term real-time monitoring of bioluminescence emitted from the kidney of semi-freely moving *Per2*^*Luc*^ mice, diurnal changes in internal environment—including body temperature and blood-borne oxygen levels—may have generated bioluminescence rhythms, independently of clock gene expression. To eliminate this possibility, we monitored bioluminescence of a circadian gene expressed at a different phase than *Per2*. Thus, this was done by analyzing mice carrying an enhanced green-emitting luciferase (ELuc), the expression of which was driven by the promoter/enhancer of the *Bmal1* gene^12^. As with *Per2*^*Luc*^ mice, clear circadian rhythms in bioluminescence were detected by real-time counting of photons emitted from the kidney of semi-freely moving *Bmal1-Eluc* mice over a period of several days (Fig. 2a, top). Circadian phases of bioluminescence data obtained from three individual mice were calculated by cosine fitting (Fig. 2a, bottom). Regression lines indicate constant circadian period length over the measurement period, and the average period length in these mice was approximately 23.5 hours, slightly shorter than in *Per2*^*Luc*^ mice. This period difference might be due either to subtle differences in genetic background between the two strains or to the effects of PER2::LUC protein fusion on the function of PER2.

The phase of expression rhythms in the endogenous *Per2* gene is known to be anti-phasic to that in the *Bmal1* gene in multiple organs *in vivo*, including the kidney^22^. Consistently with these previous findings, comparison of bioluminescence data obtained from *Per2*^*Luc*^ and *Bmal1-Eluc* mice revealed clear anti-phasic bioluminescence rhythms (Fig. 2b), eliminating the possibility that the detected bioluminescence rhythms were driven by diurnal changes in internal environment independent of clock gene expression. To confirm that PMTs detected only photons emitted from the luciferase-catalyzed enzymatic reaction, we performed photon-counting using *Bmal1-Eluc* mice continuously supplied with plain saline (Fig. 2c) and wild-type mice continuously supplied with luciferin-containing saline (Fig. 2d). Under both experimental conditions, only background and noise signals were detected.

To examine whether the present method enables reliable evaluation of circadian period length in peripheral clock gene expression, the average circadian period length was calculated by cosinor analysis based on seven-day bioluminescence data obtained from individual *Per2*^*Luc*^ mice of various age (Fig. 2e). Although aged mice showed a small interindividual variation, the period length was nearly 24 h independently of mouse age. To examine the effect of age on the period length, mice were divided into three groups based on their age, but no significant difference in period length was detected among these groups (Fig. 2f).

### Lithium lengthens kidney circadian period *ex vivo* at a concentration of 10 mM

**Figure 3:**
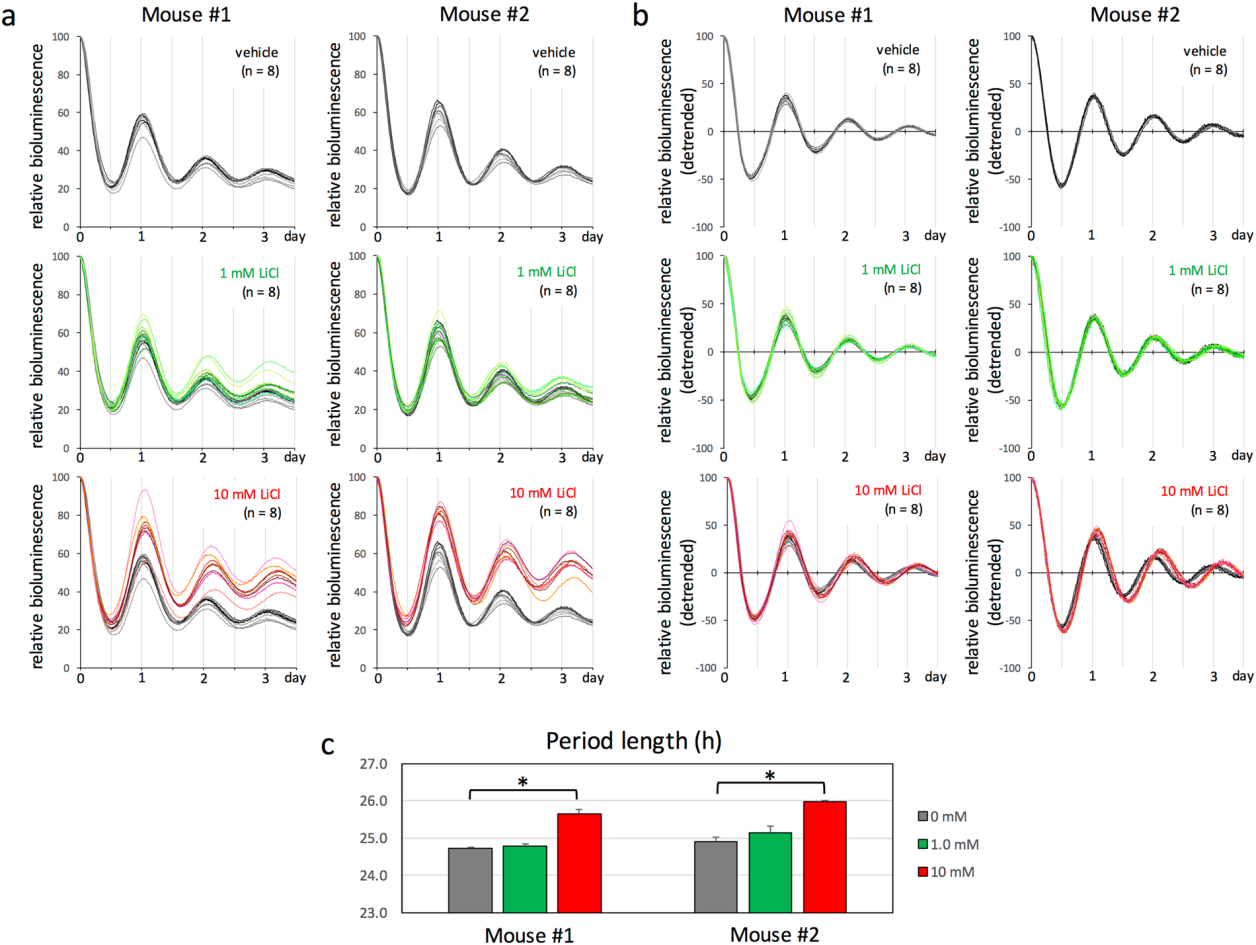
Effects of lithium on circadian period length in the kidney *ex vivo*. (**a**) Transcriptional oscillation of *Per2* was monitored as bioluminescence in kidney explants of male *Per2*^*Luc*^ mice. After synchronization by dexamethasone treatment (100 nM for 1 h), bioluminescence was measured in the presence of lithium (0 mM, black; 1 mM, green; 10 mM, red curves) and integrated for 1 min at 15 min intervals. The peak value of the first curve was set to 100 (time = 0). To facilitate visual comparison of circadian characteristics, negative control curves (0 mM, black curves) are merged with the other curves. The data are from two independent experiments (Mouse #1 and #2). (**b**) The data sets in A were detrended by subtracting the 24-h moving average from the raw data. Note the difference in period length between vehicle- and 10 mM lithium-treated explants (black versus red curves). (**c**) Period length was calculated using the data sets in B by cosinor analysis, and the average ± SE (n = 8) was compared among three different concentrations of lithium in two mice. Student’s t-test was performed and asterisks indicate P < 0.01.

Before performing *in vivo* examination of the effect of lithium on clock gene expression rhythms in the kidney using our measurement system, we examined the effect of different concentrations of lithium on *ex vivo* cultured kidney tissue. Specifically, the kidney was removed from *Per2*^*Luc*^ mice, pieces of kidney tissue were cultured in the presence of different concentrations of lithium (0, 1 and 10 mM), and the circadian period length of clock gene expression rhythms was calculated and evaluated (Fig. 3a). As indicated by previous studies, while a plasma concentration of 1 mM lithium is known to be sufficient for circadian period elongation in locomotor activity rhythms *in vivo*, a medium concentration of 10 mM is required for circadian period elongation in clock gene expression rhythms of several types of cultured cells and tissues *ex vivo* or *in vitro*^4–6^. To facilitate visual comparison, detrended values are shown as scatter plots in Fig. 3b. Clear circadian rhythms of bioluminescence were detected in all samples of cultured kidney tissue independently of lithium concentrations, and lithium-induced period elongation and amplitude enhancement were visually observed at a concentration of 10 mM. A statistical analysis based on period length values obtained with cosinor analysis demonstrated that 1 mM lithium did not affect circadian period whereas 10 mM did lengthen this period in a statistically significant manner (Fig. 3c), consistently with other peripheral clocks as reported previously^4–6^. The results were reproducible among individual mice (compare mouse #1 to mouse #2). Together, 1 mM lithium, a plasma concentration sufficient for circadian period elongation in locomotor activity rhythms *in vivo*, is insufficient to lengthen the circadian period of clock gene expression rhythms in the kidney *ex vivo*.

### 1 mM lithium lengthens circadian period length of clock gene expression in the kidney *in vivo*

**Figure 4:**
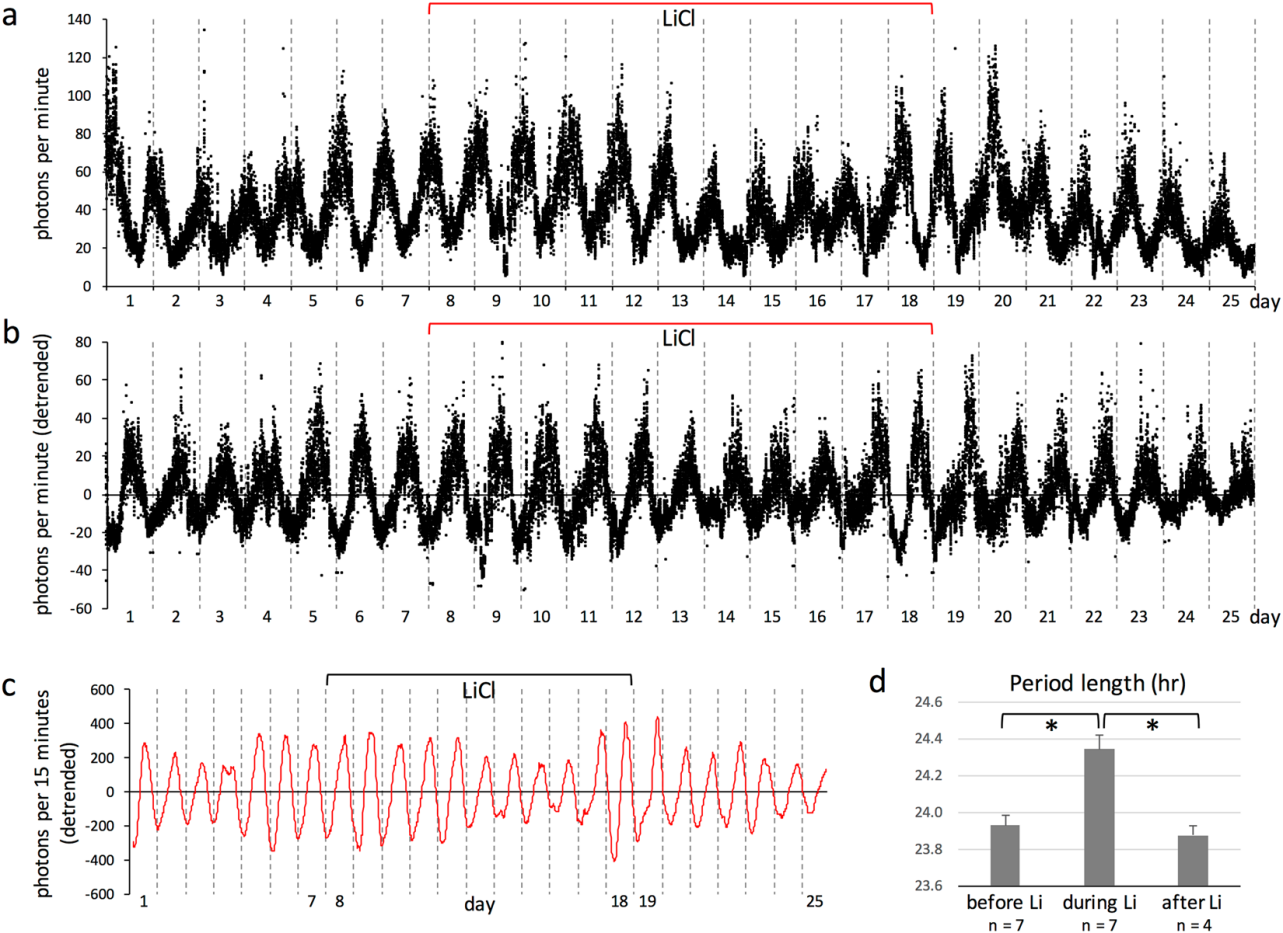
Effects of lithium on circadian period of peripheral clock gene expression *in vivo*. (**a**) Representative data of bioluminescence obtained from a *Per2*^*Luc*^ mouse that received lithium-containing water. Circadian gene expression in the kidney was monitored in real time as bioluminescence with the PMT method, for periods of 6–7 days before administration of lithium, 10–11 days during treatment with lithium and 6–7 days after removal of lithium. Photon counts were integrated for 1 min at intervals of 1 min. Lithium chloride was dissolved in drinking water of *Per2*^*Luc*^ mice at the concentration of 10 mM. Average water consumption (per mouse per day) before and during lithium administration was 2.50 ml and 3.52 ml while average food consumption was 3.82 g and 3.78 g, respectively. Seven independent experiments were performed using seven mice. (**b**) The data sets in (**a**) were detrended by subtracting the 24-h running average. (**c**) The detrended photon counts in (**b**) were integrated for 15 min at intervals of 15 min for subsequent cosinor analysis. (**d**) Circadian period length in each of these three experimental periods (6–7 days before administration, before Li; 10–11 days during treatment, during Li; 6–7 days after removal, after Li) was calculated and evaluated by cosinor analysis. Period length before or during treatment was 23.93 ± 0.05 or 24.34 ± 0.08 h (SEM, n = 7 mice), respectively. The extended circadian period of clock gene expression returned to the original following the removal of lithium from drinking water (23.88 ± 0.05 h, SEM, n = 4 mice). Asterisks indicate a significant difference (t test, P < 0.01). The number of replicates for “after Li” is smaller than that for the other experimental periods because of technical difficulty in long-term measurement.

The method for real-time monitoring of bioluminescence that we established in this study enables the simple and low-cost evaluation of circadian period length in peripheral clock gene expression with high time resolution *in vivo*. As mentioned above, although it is known that high doses of lithium induce circadian period elongation in clock gene expression under *ex vivo* and *in vitro* culture conditions, lengthening of molecular rhythms in response to lithium have not, yet, been documented *in vivo*. Accordingly, being able to detect the pharmacological efficacy of lithium via modification of circadian function *in vivo*, particularly in peripheral clocks, is a valuable asset that can provide a better understanding of its therapeutic mechanisms. We therefore used our monitoring method to examine the *in vivo* effect of lithium on circadian period length of clock gene expression in the mouse kidney (Fig. 4). Lithium chloride was dissolved in drinking water of *Per2*^*Luc*^ mice at a concentration of 10 mM. Previous reports indicate that this administration method produces plasma lithium concentrations of approximately 1 mM. Average water consumption before and during lithium administration was 2.50 ml/day and 3.52 ml/day while average food consumption was 3.82 g/day and 3.78 g/day, respectively. Thus, lithium administration had no substantial effect on either water or food consumption.

Circadian gene expression in the kidney was monitored in real time as bioluminescence for periods of 6–7 days before administration of lithium, 10–11 days during treatment with lithium and 6–7 days after removal of lithium (Fig. 4a–c). Circadian period length in each of these three experimental periods was calculated and evaluated by cosinor analysis (Fig. 4d). Period length before administration was 23.93 ± 0.05 h (SEM). In contrast, period length during treatment was 24.34 ± 0.08 h (SEM), clearly indicating that lithium, whose plasma concentrations were much lower than those in culture experiments, significantly lengthens the circadian period length of clock gene expression by 24.86 minutes *in vivo*. This extension of period length was slightly longer than but similar to that previously reported in the locomotor activity of wild-type C57/BL6 mice^5^, suggesting that both the circadian period of clock gene expression and locomotor activity are lengthened by lithium in a synchronized manner. In addition, our data demonstrated that the extended circadian period of clock gene expression gradually returned to the original following the removal of lithium from drinking water (23.88 ± 0.05 h, SEM), clearly indicating that this effect of lithium was reversible.

### *In vivo* monitoring of gene expression in the liver using the PMT method

**Figure 5:**
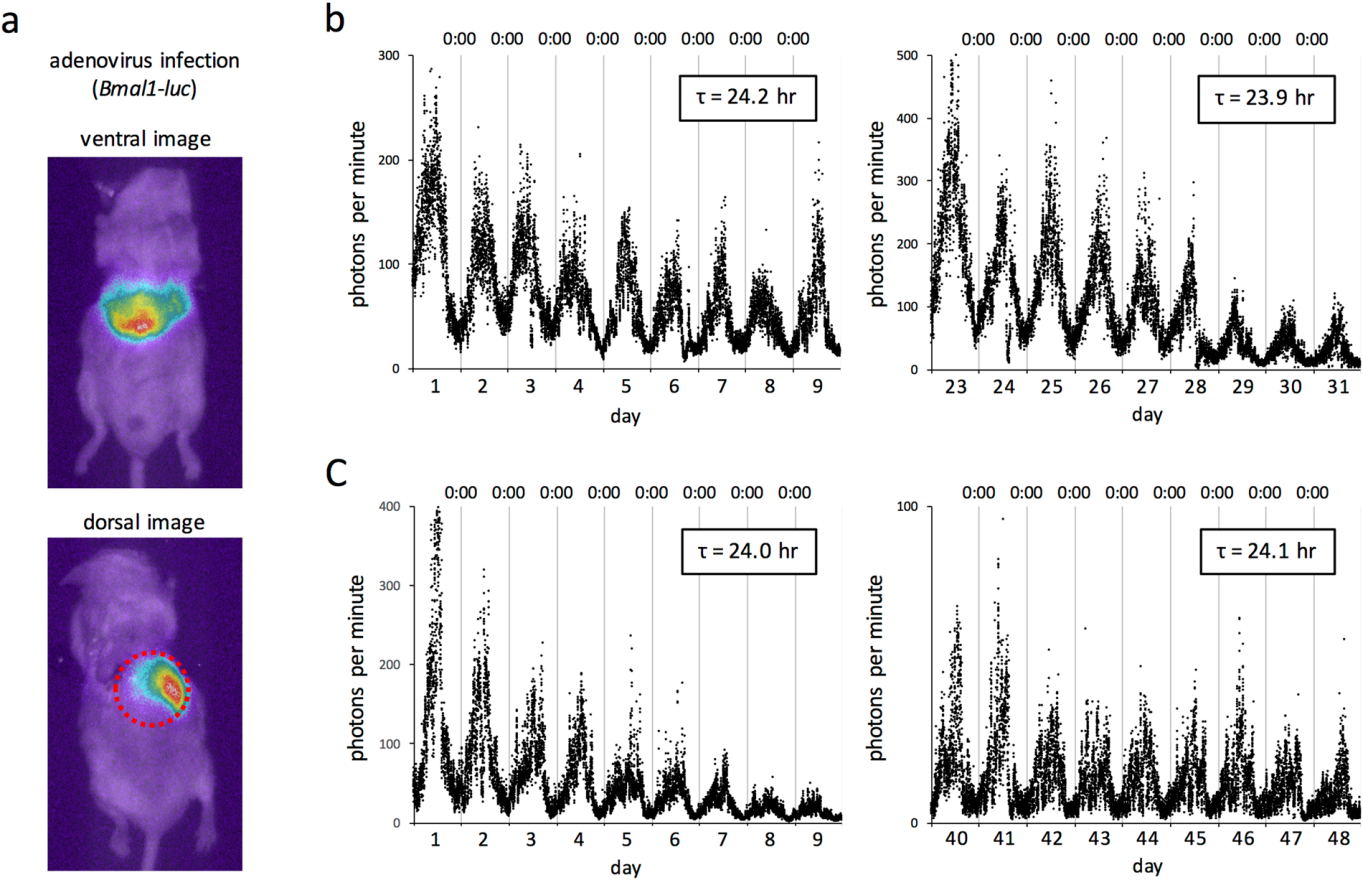
*In vivo* monitoring of circadian rhythms in clock gene expression in the liver. (**a**) Successful luciferase expression in mice was confirmed using an *in vivo* imaging technology 24 h after adenoviral infection by tail vein injection. The *luciferase* gene was driven by the transcription-regulatory region of the human *Bmal1* gene. Bioluminescence images were overlaid with bright-field images. The upper and bottom panels are a representative ventral and dorsal image, respectively. Bioluminescence intensity is shown as pseudocolor. The red dashed circle indicates a contact surface with the photon input window of the PMT. (**b**,**c**) *In vivo* real-time monitoring of bioluminescence in the liver was performed under constant dark conditions by tightly attaching the PMT to the body surface of adenovirus-infected mice, immediately after confirmation of luciferase expression by *in vivo* imaging. Mice were able to access food and water *ad libitum* during bioluminescence measurement. A syringe pump was used for continuous subcutaneous supply of luciferin. The data in (**b**,**c**) are from two different mice, and the left and right panels show data in the early and latter period of measurement, respectively. τ represents the average period length calculated by cosinor analysis using nine-day data.

As thus far described, the present method was used for real-time monitoring of gene expression in the kidney. However, the method was also expected to be theoretically applicable to other organs located in the chest and abdomen. To examine this possibility, we performed *in vivo* real-time monitoring of bioluminescence using mice infected with an adenovirus carrying the *luciferase* gene whose expression was driven by the transcription-regulatory region of the *Bmal1* gene. This experimental approach enables real-time monitoring of *Bmal1* expression as bioluminescence in a liver-specific manner because it is well known that an adenovirus introduced into mice by tail vein injection infects the liver. In fact, liver-specific bioluminescence signals were detected in both ventral and dorsal images by *in vivo* imaging (Fig. 5a).

As with the kidney, a small PMT was fixed on the back of an infected mouse, and circadian rhythms in clock gene expression in the liver were monitored in real-time. The liver is anatomically located slightly superior to the kidney, and the position of the PMT was therefore adjusted based on bioluminescence images. Figure 5b,c show circadian bioluminescence obtained from two mice. The data indicate that *Bmal1* gene activity reached maximum a little before noon, as is the case with the kidney in Fig. 2a. This is consistent with the fact that the transcripts of endogenous clock genes, including *Bmal1*, peak simultaneously in both the kidney and liver^23^. Because transgenes delivered by adenovirus vectors are transiently expressed but not integrated into the host genome, we were concerned that they are not suitable for long-term monitoring of gene expression. However, circadian bioluminescence did not attenuate even three weeks after infection (Fig. 5b, right), and a similar circadian period length in bioluminescence was detectable six weeks after infection, albeit with a decrease in signal intensity (Fig. 5c, right). Adenovirus vectors are therefore useful for the long-term monitoring of gene expression. Together, these results indicate that this method is applicable to gene expression monitoring in various organs located in the abdomen - and probably also in the chest - by tissue-specific expression of reporter genes.

## Discussion

### Effect of lithium on circadian system

Circadian dysfunction is considered to play a role in the pathogenesis and progression of human bipolar disorders^2,1^. Lithium has been used as a mood stabilizer to treat human bipolar disorders for over half a century, but the mechanism by which lithium exerts its mood stabilizing effects remains to be determined. Several studies have suggested the possibility that the efficacy of lithium treatment results in part from the modification of circadian function^3^. One piece of evidence for this putative action of lithium on circadian rhythms is that lithium administration lengthens the circadian period in behavior and physiology from unicellular organisms to humans under constant environmental conditions, such as constant darkness. Although it is unclear how this period elongation effect leads to symptom amelioration of bipolar disorders, these findings at least suggest that lithium potentially acts to target and adjust circadian rhythms *in vivo*. In addition to this *in vivo* effect, administration of lithium to cultured cells and tissues causes circadian period elongation in clock gene expression rhythms^4–6^, suggesting that lithium directly acts on cell-autonomous core circadian machinery *in vitro* and *ex vivo*. Many studies have consistently reported that glycogen synthase kinase 3β (GSK3β), a kinase whose activity is modified by lithium^24^, phosphorylates circadian clock proteins and regulates their function and stability^25–28^.

The effect of lithium on clock gene expression rhythms of cultured cells and tissues is thus consistent with that on circadian rhythms in behavior: lithium lengthens both rhythms. Presently, however, it remains unproven whether lithium causes circadian period elongation in clock gene expression rhythms *in vivo*. In this context, a significant question is why the medium concentrations of lithium required for circadian period elongation in clock gene expression rhythms *in vitro* and *ex vivo* (10 mM and more) are much higher than the plasma concentrations required for period elongation in behavioral rhythms *in vivo* (less than 1 mM)^5^. Consistent with this, we found that 1 mM lithium was insufficient to lengthen the circadian period of clock gene expression rhythms in the kidney *ex vivo* (Fig. 3). Given that the therapeutically effective concentration of lithium in serum (approximately 0.5–1.5 mM) is lower than the IC50 of lithium for the inhibition of GSK3β (approximately 2 mM)^29^, inhibition of GSK3β by lithium at therapeutic levels may also be insufficient to contribute significantly to mood stabilization. Although a few reports indicated that low doses of lithium (1–2 mM) lengthen circadian period elongation in the suprachiasmatic nucleus (SCN) under *ex vivo* culture conditions^4,30^, this conclusion remain controversial^5^. The effect of lithium on core-clock gene expression, particularly of peripheral clocks, has not yet been investigated *in vivo* because, as already explained, detection of changes in period length *in vivo* using traditional experimental approaches is technically difficult. To overcome this technical limitation, in the present study, we developed a simple and low-cost method for real-time monitoring of clock gene expression rhythms in the kidney and liver *in vivo*. Using this method, we clearly found that lithium causes circadian period elongation in clock gene expression rhythms *in vivo*. Our data also indicate that the extended period length gradually returns to the original after removal of lithium from drinking water: in other words, the period elongation effect of lithium is reversible.

Our present data showed that the effect of lithium on circadian period length in peripheral circadian gene expression was completely reversible. This is inconsistent with a previous report that showed that the effect in locomotor activity was incompletely reversible, with the period length remaining significantly longer than the original even four weeks after removal of lithium^5^. Two reasons may explain this inconsistency. First, lithium-induced aftereffects in the previous study may have been stronger than those in our experiments because the former examined the effects of more than 30 days exposure to lithium compared to 10–11 days in the latter. Therefore, it would have taken longer to eliminate the aftereffects in the previous study. Interestingly, chronic exposure of mice to light and dark cycles with a period shorter or longer than 24 h induces prolonged aftereffects in circadian period length via epigenetic modifications in the SCN^31^. It is possible that similar biological processes may occur in the SCN of mice receiving lithium for a long period of time because lithium imposes a non-24 h circadian period on mice. Second, although brain and blood concentrations of lithium are similar^32^, a few reports, as described above, have indicated that the SCN clock is more sensitive to lithium than peripheral clocks^4,30^, suggesting that a longer period of time is needed to eliminate the effect of lithium on the SCN clock compared to peripheral clocks. The incomplete reversibility of the effect of lithium on circadian period length in locomotor activity observed in the previous report may be due to prolonged aftereffects on the SCN clock.

Although in this study we only focused on the *in vivo* effect of lithium on the kidney, as a representative peripheral clock, it is likely that similar lithium-induced period lengthening occurs in clock gene expression of other peripheral clocks, as well as on the SCN. However, it cannot be excluded that other organs and tissues may exhibit only minor, or negligible, period lengthening *in vivo*, on the basis of a report which indicated that the magnitude of the effect of lithium on clock gene expression varies among organs and tissues *ex vivo*^5^. Given that low doses of lithium do not affect circadian period in peripheral clock gene expression *in vitro* or *ex vivo*, some different mechanisms from those *in vitro* and *ex vivo* may be involved in lithium-induced period elongation in clock gene expression rhythms *in vivo*. As potential mechanisms, we consider two cases. First, a factor or factors which enhances lithium efficacy exists *in vivo*, explaining that low doses of lithium do not exert circadian period elongation activity because of the lack of the factor(s) under culture conditions. Second, lithium lengthens circadian period *in vivo* not via cell-autonomous regulation, but rather by unknown systemic regulation.

### Potential application of PMT method

Biochemical or histochemical measurement of gene expression in animal organs with high time resolution over periods of a few weeks is markedly difficult, and we are unaware of any method that enables this, particularly in a simple and low-cost manner. Although some optical monitoring approaches using transgenic mice carrying a reporter gene are therefore required, existing measurement systems are currently expensive and complicated to implement. In the present study, we developed a method for continuous real-time measurement of gene expression in semi-freely moving mice over periods of up to six weeks, which involves the simple attachment of a small PMT tightly onto the body surface. Advantages of this system include the simplicity of manipulation, the low invasiveness, the lack of requirement of continuous anesthesia, and the automated nature of measurement. Further optimization is required to increase the success rate of long-term measurement. Our detection unit is small and inexpensive and therefore expandable to a high-throughput platform. The present study focused on clock gene expression, but the method is applicable to real-time detection of the expression of a wide range of genes.

The PMT method overcomes weak points in the measurement systems reported previously, but still has some limitations. First, the PMT method has a disadvantage in tissue-specific monitoring of gene expression. However, the use of tissue-specific promoters to express a reporter gene enables tissue-specific monitoring with the PMT method in a more precise manner, and the use of bright luminescent reporter proteins enables the measurement of photons from deep-lying organs. Second, although not detected in this study, continuous luciferin administration may cause unexpected secondary effects on other biological processes. Third, although a strongpoint of our PMT method is that it enables the long-term overview of gene expression in a highly time-resolved manner with only small number of mice and without killing them, the data obtained show only relative changes in expression levels. While this limitation did not affect circadian phase and period evaluation in the present study, the drawing of any firm conclusions on quantitative changes such as circadian amplitude may require traditional quantitative biochemical analyses.

Further development of our detection system to maturity will aid a wide range of research fields in medicine and biology. In addition to mechanical improvements, development might also extend to enhancing bioluminescence intensity and the use of tissue-specific promoters, which would help expand the application of the system. Among examples, our system may help investigate developmental stages in which a target gene is expressed without killing a large number of pregnant mice. This simple long-term monitoring method will also be powerful in examining the chronic effect of environmental stimuli on expression of a target gene in organs. In particular, our method may greatly aid in the development of new medicines. When performed with traditional methods, long-term monitoring of the impact of drug treatment on target gene expression with high time resolution requires vast numbers of mice. However, our method offers marked advantages in throughput performance and would therefore be useful even for large-scale experiments, such as the screening of drugs that affect target gene expression. Finally, our method may accelerate research on circadian rhythms. While biochemical examination of circadian gene expression in organs around the clock is a standard experiment in this field, long-term overnight organ sampling imposes a severe burden on researchers. Our method will help researchers deal with this issue.

## Supplementary information

Figure S1
