# Supplementary material for: In vivo evaluation of the effect of lithium on peripheral circadian clocks by real-time monitoring of clock gene expression in near-freely moving mice
Source: Sci Rep. 2019 Jul 29;9:10909. doi: 10.1038/s41598-019-47053-3 (PMC6662689; doi:10.1038/s41598-019-47053-3)
Supplement: Supplementary file 1 — Figure S1 [file 41598_2019_47053_MOESM1_ESM.pdf]

Supplementary information

In vivo evaluation of the effect of lithium on peripheral circadian clocks by real-time monitoring of clock gene expression in near-freely moving mice

Yuka Sawai[1], Takezo Okamoto[1], Yugo Muranaka[1], Rino Nakamura[1], Ritsuko Matsumura[1], Koichi Node[2] and Makoto Akashi[1, *]

[1]The Research Institute for Time Studies, Yamaguchi University,
1677-1 Yoshida, Yamaguchi 753-8511, Japan
[2]Department of Cardiovascular Medicine, Saga University,
5-1-1 Nabeshima, Saga 849-8501, Japan

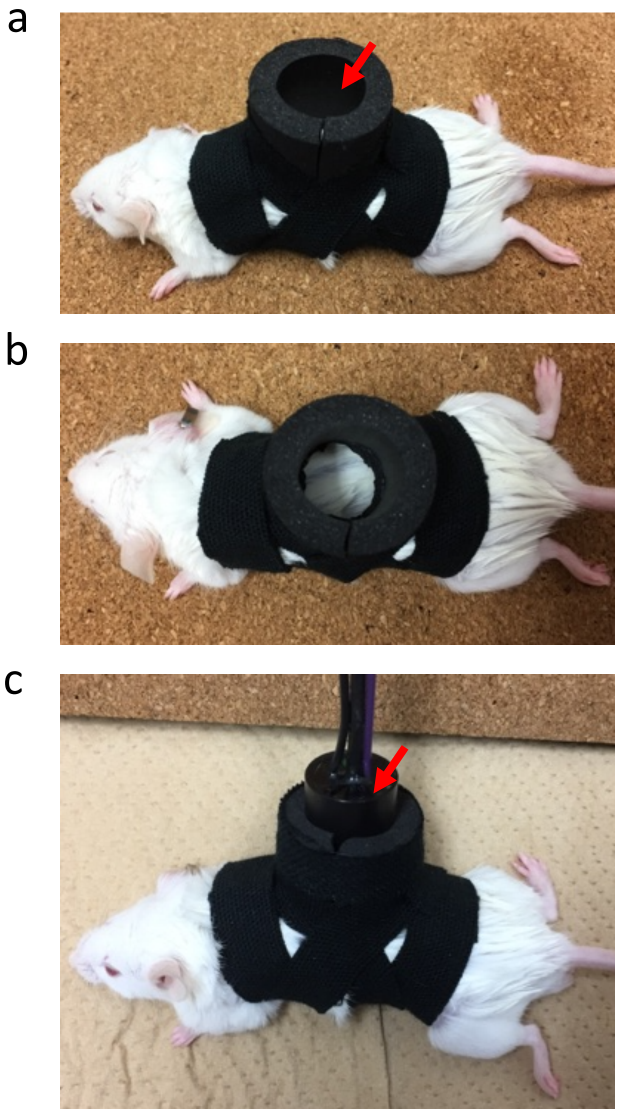

Figure S1

How to fix a PMT on the mouse's back

(a) An open cylinder made of sponge-like material (red arrow) was fixed as a socket for a PMT on the mouse's back with an elastic medical band. (b) The kidneys are localized around the lowest rib on the back, where the center of the cylinder was set. (c) A small PMT (red arrow) was simply inserted into the cylinder.
